# Photoswitchable optoelectronic properties of 2D MoSe_2_/diarylethene hybrid structures

**DOI:** 10.1038/s41598-024-57479-z

**Published:** 2024-03-27

**Authors:** Sewon Park, Jaehoon Ji, Connor Cunningham, Srajan Pillai, Jean Rouillon, Carlos Benitez-Martin, Mengqi Fang, Eui-Hyeok Yang, Joakim Andréasson, Jeong Ho You, Jong Hyun Choi

**Affiliations:** 1 School of Mechanical Engineering, Purdue University, West Lafayette, IN 47907 USA; 2 Department of Mechanical Engineering, University of St. Thomas, St. Paul, MN 55105 USA; 3 Department of Chemistry and Chemical Engineering, Chalmers University of Technology, 412 96 Gothenburg, Sweden; 4 Department of Chemistry and Molecular Biology, University of Gothenburg, 413 90 Gothenburg, Sweden; 5 Department of Mechanical Engineering, Stevens Institute of Technology, Hoboken, NJ 07030 USA

**Keywords:** Transition metal dichalcogenide, MoSe_2_, Optoelectronics, Photoswitch, Photochromic molecules, Diarylethene, Two-dimensional materials, Nanoscale devices

## Abstract

The ability to modulate optical and electrical properties of two-dimensional (2D) semiconductors has sparked considerable interest in transition metal dichalcogenides (TMDs). Herein, we introduce a facile strategy for modulating optoelectronic properties of monolayer MoSe_2_ with external light. Photochromic diarylethene (DAE) molecules formed a 2-nm-thick uniform layer on MoSe_2_, switching between its closed- and open-form isomers under UV and visible irradiation, respectively. We have discovered that the closed DAE conformation under UV has its lowest unoccupied molecular orbital energy level lower than the conduction band minimum of MoSe_2_, which facilitates photoinduced charge separation at the hybrid interface and quenches photoluminescence (PL) from monolayer flakes. In contrast, open isomers under visible light prevent photoexcited electron transfer from MoSe_2_ to DAE, thus retaining PL emission properties. Alternating UV and visible light repeatedly show a dynamic modulation of optoelectronic signatures of MoSe_2_. Conductive atomic force microscopy and Kelvin probe force microscopy also reveal an increase in conductivity and work function of MoSe_2_/DAE with photoswitched closed-form DAE. These results may open new opportunities for designing new phototransistors and other 2D optoelectronic devices.

## Introduction

Two-dimensional (2D) transition metal dichalcogenides (TMDs) have gained significant attention in recent years due to their remarkable physical properties, making them attractive for nanoelectronics^1–4^. Recent advances in 2D TMDs have revealed their versatility, characterized by van der Waals interactions^5,6^, high mobility^7^, and layer-number-dependent indirect-direct bandgap transitions^8^. These unique attributes can lead to valley polarization and strong interlayer photoluminescence (PL) emissions via excitonic recombination^9^. There have been various studies to control these factors and modulate charge transport in TMDs, including gate-induced electric doping^10,11^, polarization switching^12^, and spin-valley tuning^13^. However, these conventional approaches present challenges and complexities in manufacturing and characterization processes. In the case of gate-induced control, for example, it is necessary to construct a back-gate field-effect transistor (FET) to regulate carriers, which, in turn, requires low contact resistance and high-quality contact. For polarization switching and spin-valley tuning, high magnetic fields are needed^13,14^.

On the other hand, a chemical functionalization approach constructs atomically-thin hybrid structures made of TMDs and functional organic molecules. This method, which involves charge transfer at the hybrid interface, is simple yet promising for modifying the optoelectronic properties of TMDs. For example, it includes techniques like dipping and spin-coating^15,16^. This process creates potential differences between the TMD and the chemical material, where the adsorbed chemicals change the optical and electrical properties of the underlying flakes. Applying organic layers to TMDs has been recognized as an effective means to control charge transport between the target layers^17–19^. Besides the simplicity of the procedure, it offers significant advantages, including adaptability, scalability, and noninvasive/reversible functionalization, making it a flexible and cost-effective choice for a wide range of applications^20,21^.

Diarylethenes (DAEs)^22–24^ are photoswitchable organic molecules that, alongside azobenzenes^25,26^ and spiropyrans^27^, are the most popular photochromic family. These materials respond to light stimuli; they undergo dynamic structural transformation between two or more isomeric forms upon irradiation of specific wavelengths^28,29^. Several studies have explored interactions between photochromic molecules and TMD flakes. Given that the photoswitching behavior of DAE, FETs with light-controlled on/off behaviors were demonstrated using few-layer WSe_2_/DAE^30^, by modifying charge transport at the hybrid interfaces. Similarly, azobenzenes and spiropyrans were exploited to modulate electrical signals of TMDs^31–33^. Despite these efforts, the optical characteristics in photoswitchable TMD devices and underlying theoretical principles remain largely unexplored.

This work elucidates dynamic and reversible photo-modulations of monolayer MoSe_2_/DAE hybrid structures both experimentally and theoretically. Density functional theory (DFT) calculations are used to design and understand the interfaces and photo-processes. DAE undergoes unique transformations when exposed to UV or visible light, switching between two isomers, closed and open forms. We demonstrate that its highest occupied molecular orbital (HOMO) and lowest unoccupied molecular orbital (LUMO) energy levels shift during the transformation and that the photoswitch can be exploited for modulating optoelectronic properties of monolayer MoSe_2_. We also show that PL intensities are modulated drastically by alternating UV and Vis light irradiation, which may be cycled repeatedly. Conductive atomic force microscopy (C-AFM) and Kelvin probe force microscopy (KPFM) confirm the light-controlled charge transfer at the hybrid interface. Our findings will provide critical insights into fundamental properties in 2D semiconductors and offer new opportunities for photoswitchable hybrid devices.

## Materials and methods

### Preparation of MoSe_2_

P-doped SiO_2_/Si substrates were used for the sample preparation. These substrates underwent sequential ultrasonication in the following order: acetone, methanol, and deionized water (DI), with each solvent treatment lasting 15 min. Subsequently, the substrates were subjected to forced air drying to remove residual fluids and then placed on a hot plate at 110 °C for 5 min. The mechanical exfoliation from bulk crystals (2D semiconductor) was applied to obtain MoSe_2_ flakes, as described previously^17,34^. Briefly, Scotch magic tape was used to affix the exfoliated flakes for 10 min on a hot plate at 50 °C, and they were then compressed for an additional 10 min. Similarly, samples were also prepared on indium tin oxide glass (ITO, MTI KJ Group) substrates with the hot plate treatment extended to 30 min. After tape removal, each sample was subjected to annealing under Argon gas for an hour to improve interfacial contact and eliminate contaminants^19,35^.

### Preparation of DAE molecules

DAE was prepared according to a modified reference procedure^36^. Knochel modification^37^ was applied to the Negishi coupling 2,4-dibromo-5-methylthiazole and 3,5-bis(trifluoromethyl)-1-bromobenzene for the obtention of 2-(3,5-bis(trifluoromethyl)phenyl)-4-bromo-5-methylthiophene. This intermediate was then transformed in situ to the corresponding boronic pinacol ester, necessary for the Suzuki coupling with 1,2-dichlorohexafluorocylopent-1-ene that gives access to the desired DAE. All experimental details are thoroughly described in the Supplementary Information (SI Sect.  S1).

### Functionalization of MoSe_2_ with DAE

DAE solutions were prepared in chloroform at approximately 0.02 mg/mL. Exfoliated MoSe_2_ samples on silicon wafer and ITO glass substrates, were immersed in the DAE solution for 12 h at 4 °C, forming a uniform layer of DAE on MoSe_2_^30^. Afterward, the excess DAE solution on the substrate was removed using an air gun, and the samples were soft-baked on a hot plate at 50 °C for 30 min. The deposited DAE molecules demonstrate a uniform film on MoSe_2_ flakes, while their exact orientation may be affected by several factors including steric interaction. To prevent unintended light exposure, the samples were stored in a dark room before UV and visible light irradiation. To investigate the photoswitching behavior, MoSe_2_/DAE samples were exposed to UV for 2 min and visible light for 10 min (see SI Sect. S2 and Fig. S3)^30^. UV light was generated using a Spectroline™ TE lamp (312 nm, 8W), while visible light was filtered through a 530 nm long-pass filter on a Xe-arc lamp with a power intensity of ~ 7.7 mW/cm^2^, monitored using a Newport power meter.

### DFT calculation

Vienna Ab initio Simulation Package (VASP) was employed for DFT calculations, utilizing the projector augmented wave (PAW) method^38,39^. Electron exchange and correlation were treated with the generalized gradient approximation in the form of Perdew-Burke-Ernzerhof (PBE) functional^40^. The electronic minimization was performed with a tolerance level of 10^–5^ eV. To prepare DAE-MoSe_2_ hybrid systems, a DAE isomer was placed above the (5 × 5) MoSe_2_ monolayer slab (see Fig. S5) and a vacuum of 14 Å was added. The corresponding areal density is 4.2 × 10^13^ molecule/cm^2^. To properly describe van der Waals interactions between the MoSe_2_ monolayer and DAE, a dispersion correction was added using the DFT-D2 method of Grimme^41^. All ionic positions were relaxed until Hellmann–Feynman forces were less than 10 mV/Å with a 2 × 2 × 1 Γ-centered **k**-point mesh for the DAE-(5 × 5) MoSe_2_ hybrid systems. After the ionic relaxation, a finer **k**-point mesh of 4 × 4 × 1 Γ-centered mesh was used for generating accurate density of states.

### Optical characterization

The Raman and optical measurements were conducted using a Renishaw inVia Raman confocal microscope at room temperature. Flake inspection was conducted with a charge-coupled device camera (CCD) to find monolayer MoSe_2_. The experiments were conducted using 10 ×, 20 ×, 50 ×, and 100 × objective lenses to identify the locations of flakes on the substrates. PL measurements for the flakes were carried out using a 785 nm laser (~ 0.1 mW) and a 100 × objective lens, thus avoiding the absorbance range of DAE while inducing photo-processes in MoSe_2_. Note that the PL distribution does not follow a Gaussian distribution, especially at high energies (or under 790 nm). This is because the MoSe_2_ emission is close to the excitation energy (785 nm) which is removed using a notch filter. However, the measured emission peak at ~ 795 nm is consistent with other studies^42,43^, suggesting that this effect may be negligible in characterizing photo-modulations.

### AFM, C-AFM, and KPFM characterization

The exfoliated flakes and DAE-functionalized samples were examined with Bruker Dimension Icon AFM. A SCANASYST-AIR probe was employed in topological imaging to determine the flake thickness, while a SCM-PIT-V2 probe coated with Pt-Ir was used for C-AFM and KPFM measurements. For the C-AFM and KPFM measurements, samples were prepared on ITO glass substrates. In C-AFM, a current range was established within ± 12 nA with a sensitivity of 1 nA V^−1^ to prevent potential damage to both the tip and the sample while applying a bias voltage from − 2 to 2 V^17,19^.

## Results and discussion

**Figure 1:**
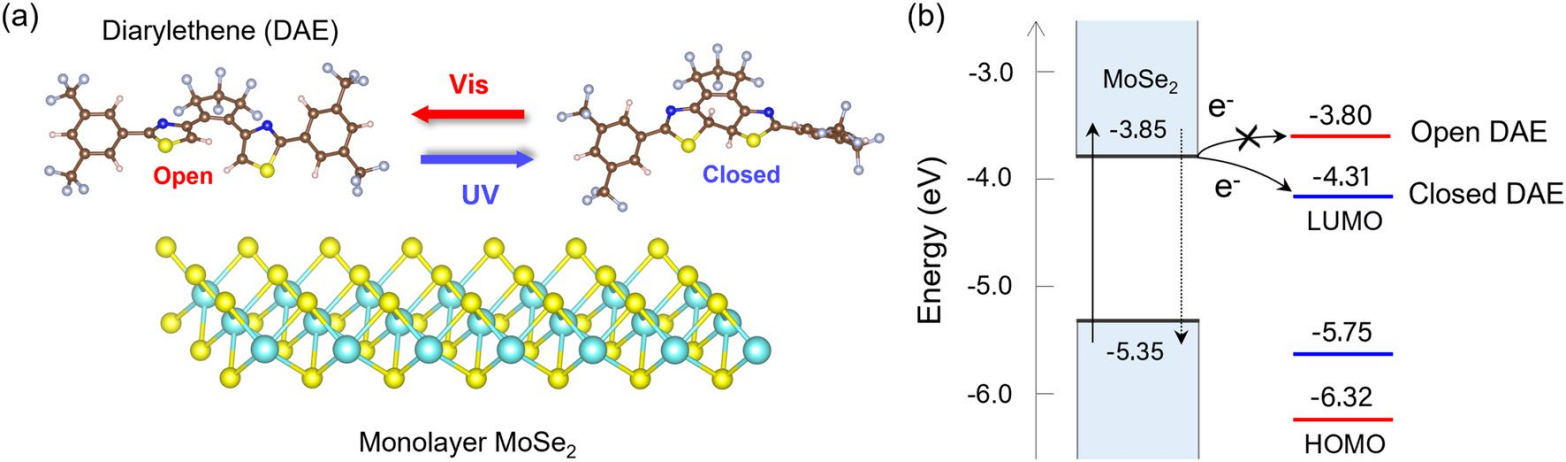
Schematic and energy diagram of a hybrid structure from monolayer MoSe_2_ and DAE. (**a**) Irradiation of UV and visible (Vis) light switches the molecular structure of DAE, transitioning between π-conjugated closed and cross-conjugated open forms. (**b**) Alignment of the VBM and CBM of monolayer MoSe_2_ against the HOMO and LUMO energy levels of open- (Vis) and closed-form (UV) isomers of DAE. The HOMO and LUMO levels of open-form DAE are lower and higher than the VBM and CBM of MoSe_2_, respectively. In contrast, the closed-form has its LUMO level lower than the CBM, while its HOMO level remains lower than the VBM. As a result, photoexcited electron transfer may be energetically favored from MoSe_2_ to closed-form DAE, whereas it will be forbidden between MoSe_2_ and open-form DAE. Overall, photoswitching can regulate charge transfer between MoSe_2_ and DAE.

Figure 1a presents the isomerization scheme for the DAE derivative used in this study. The open isomer absorbs exclusively in the UV region, whereas the closed isomer displays absorption also in the visible spectrum (Fig. S3). We hypothesize that the photoswitching behavior of DAE molecules would be retained within deposited layers on top of TMD flakes. This is a reasonable assumption given previous reports that included several photochromic molecules interfacing TMDs as discussed above.

In this work, the photoswitchability of DAE molecules is designed to engineer charge transfer mechanisms in MoSe_2_/DAE and thereby modulate the optoelectronic properties of the TMD monolayers. UV and visible irradiation will thus control the exciton formation and behavior. Our scheme is illustrated in Fig. 1b, where the valence band maximum (VBM) and conduction band minimum (CBM) of monolayer MoSe_2_ are plotted against the two sets of HOMO and LUMO energy levels corresponding to the open and the closed DAE photoisomers. Both MoSe_2_ and DAE energies were obtained from DFT calculations (vide infra) which are in excellent agreement with previous report^44^. The CBM and VBM energies against the vacuum level are approximately − 3.85 and − 5.35 eV, respectively. The energy levels are consistent with previously reported data^45,46^. The LUMO and HOMO levels of open-form DAE are approximately − 3.80 eV and − 6.32 eV, while − 4.31 eV and − 5.75 eV for closed-form isomers.

Notably, the LUMO level of closed-form DAE is below the CBM of monolayer MoSe_2_, whereas that of the open-form is above the CBM. Both isomers have their HOMO energies below the VBM. This suggests the feasibility of the following scenario. The photoexcitation in a MoSe_2_ flake will create excitons, excited electrons in the conduction band and holes in the valence band. The electrons may be transferred to the LUMO of the closed-form DAE, but not the open-form. The hole transfer to either form is energetically not favorable. Therefore, it is possible to facilitate the photoexcited electron transfer by irradiating UV (to form closed DAE) or suppress it with visible light (to form open-DAE). As a result, UV irradiation will reduce the radiative recombination of photoexcited electron–hole pairs, resulting in a PL quenching^16,47^. Visible illumination will isomerize the DAE into the open-form and recover the emission properties. This mechanism should be reversible and fast, occurring on the picosecond timescale^22,48^.

**Figure 2:**
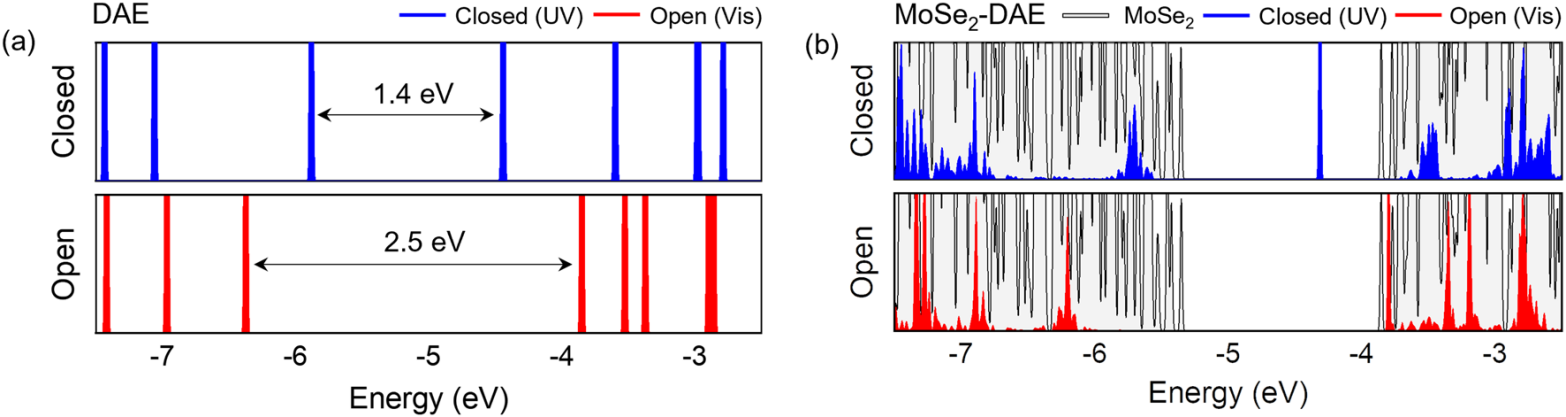
DFT-calculated density of electronic states (DOS) of (**a**) a DAE molecule and (**b**) a DAE-MoSe_2_ hybrid. (**a**) DOS of closed- and open-forms of DAE, highlighting a shift of the LUMO and HOMO energies through photoisomerization. The vacuum level is set to zero. The HOMO and LUMO levels of the closed-form (under UV irradiation) are approximately − 5.75 eV and − 4.31 eV, respectively; for the open-form (irradiated by visible light), they are about − 6.32 eV and − 3.80 eV. (**b**) In the hybrid DAE-MoSe_2_ monolayer, the LUMO level of the closed isomer becomes lower than the MoSe_2_ CBM, while it is higher with the open-form. This photoswitchability of DAE provides available states within the bandgap for photoinduced electron transfer from MoSe_2_ to closed-form DAE (but not to open-form) and allows for a photo-modulation of optoelectronic properties of monolayer MoSe_2_.

The energies of monolayer MoSe_2_, DAE, and hybrid MoSe_2_/DAE were obtained from DFT calculations using VASP^38,39^. Figure 2a presents the electronic states of DAE isomers with the vacuum level set as zero. The transformation from the closed isomer to the open raises the LUMO energy level and lowers the HOMO, resulting in an increase in the HOMO–LUMO gap by about 1.1 eV. The density of states (DOS) of the DAE/MoSe_2_ monolayer slab is shown in Fig. 2b. The VBM of monolayer MoSe_2_ is set as zero for comparison purposes. With the open DAE isomer, both HOMO and LUMO stay outside the bandgap of MoSe_2_. When the DAE isomerizes to the closed-form, the LUMO shifts into the bandgap, providing available states for the electrons in MoSe_2_ to transfer to. The HOMO remains outside the bandgap. Thus, there would be no hole transfer. The DFT results support our photo-modulation scheme.

**Figure 3:**
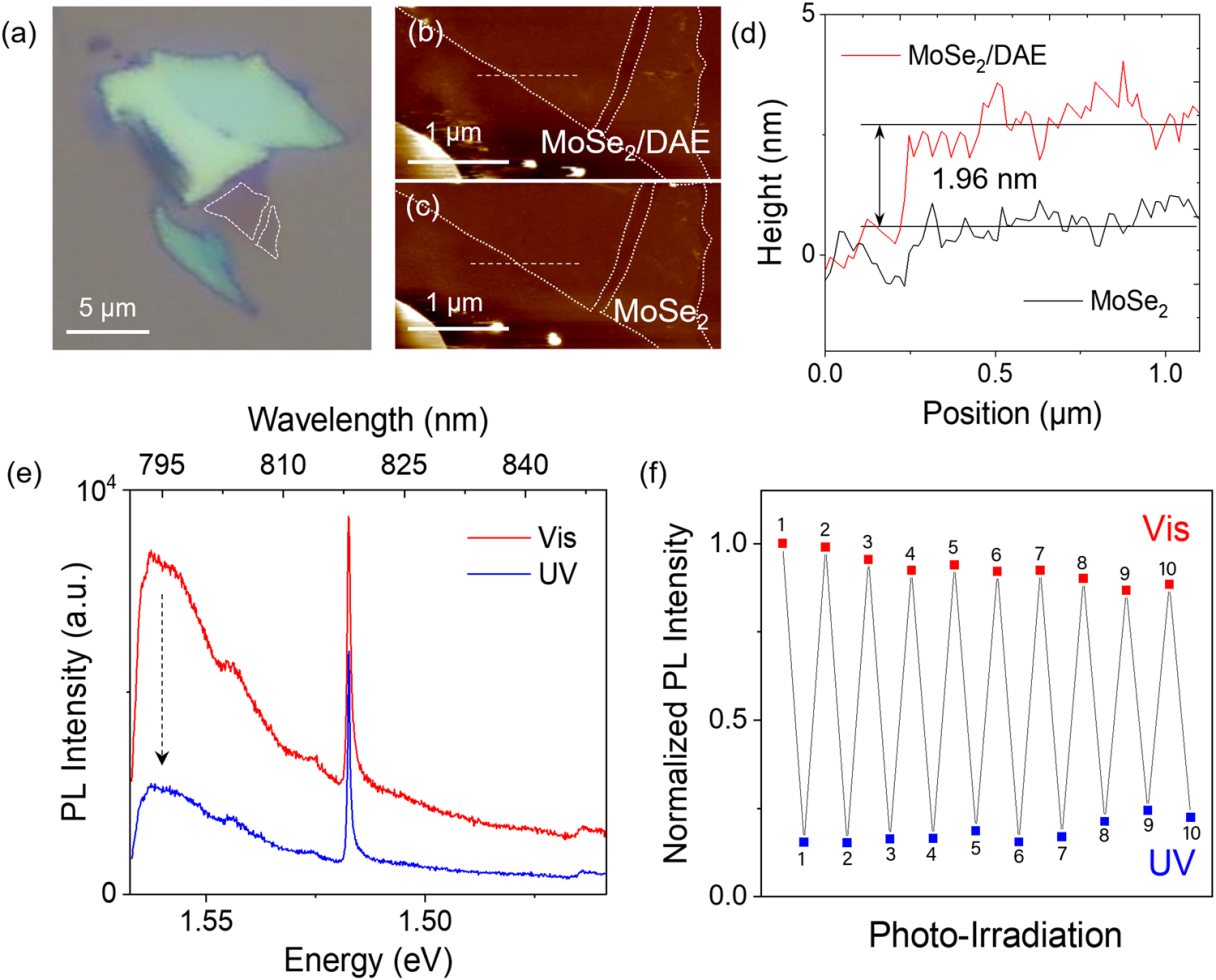
(**a**) Bright-field image of mechanically exfoliated pristine MoSe_2_ on a SiO_2_/Si substrate. AFM images of (**b**) MoSe_2_/DAE and (**c**) MoSe_2_ corresponding to the monolayer flake shown in (**a**). (**d**) Height profiles of MoSe_2_ and DAE-MoSe_2_ indicated by the dotted lines in (**b**,**c**). The thickness of the DAE layer is approximately 2 nm. (**e**) PL spectra of MoSe_2_/DAE (in linear scale) measured under a confocal Raman microscope with a 785-nm diode laser excitation. After visible and UV irradiation, the sample displays PL emission peaks at approximately 795 nm (~ 1.56 eV). The sharp feature at ~ 818 nm (corresponding to ~ 520 cm^−1^) is the Raman signature of the silicon substrate. Drastic PL quenching is observed after UV irradiation. (**f**) Cyclic measurements of the PL modulation with alternating visible and UV irradiation. Ten cycles of PL modulation demonstrate photoswitchable optoelectronic properties of monolayer MoSe_2_ with photochromic DAE.

To examine our strategy, we first investigated the PL quenching effect. MoSe_2_ flakes were prepared on a SiO_2_/Si substrate via mechanical exfoliation and annealed at 250 °C for an hour. This procedure enhances the PL intensity of the n-type MoSe_2_ by depleting excess electrons^49,50^. The samples were then immersed in a DAE chloroform solution, forming a layer of DAE molecules on MoSe_2_ (see the “Materials and methods” section). Figure 3a shows an optical image of MoSe_2_, with dotted lines marking the monolayer flake's boundary. To examine the surface and height of the MoSe_2_/DAE hybrid before and after functionalization, we conducted AFM measurements, as depicted in Fig. 3b,c. Figure 3d presents the height information derived from the AFM images, revealing the thickness of monolayer MoSe_2_ of ~ 0.8 nm and the DAE layer of ~ 2 nm. DAE has an absorption range in the visible spectrum from 450 to 650 nm (Fig. S3). To avoid the absorbance range and excite MoSe_2_ exclusively, we selected a 785 nm laser for excitation. To study the interaction of MoSe_2_ with closed- and open-form DAE, we irradiated the hybrid MoSe_2_/DAE sample with UV (~ 312 nm for 30 s) and visible light (530-nm long-pass filter for 10 min), respectively.

Monolayer MoSe_2_/DAE exhibited strong PL signature peaking at ~ 795 nm (Fig. 3e), consistent with previous reports^17,42^. A sharp feature at ~ 818 nm (equivalent to ~ 520 cm^−1^) is a Raman signature from the underlying silicon wafer. After UV irradiation, the closed-form isomers promoted the photoexcited electron transfer from MoSe_2_ to DAE, which resulted in a drastic PL quenching by ~ 80% in intensity compared to the open-form under visible light. Note that the intensity of the Raman signature did not change significantly during the PL quenching. The energetically favorable charge transfer mechanism is attributed to the closed DAE’s LUMO energy being lower than the CBM of MoSe_2_, as designed in our scheme. In contrast, the LUMO level of the open-form is higher than the CBM. Therefore, electron transfer is prohibited, and PL quenching is not observed. To test the reversibility and consistency of this photoswitching behavior, we repeatedly exposed the sample to visible and UV light for 10 cycles. The result in Fig. 3f shows remarkable stability of MoSe_2_/DAE photoswitch with a steady quenching ratio, ranging between 70 and 85%. During the cycle repetition, there is a gradual decrease in PL intensity difference between visible and UV irradiation. For example, it gradually drops to about 90% of the initial PL difference after the 10th cycle. This degradation, termed fatigue, results from the irreversible formation of byproducts during repeated switching, which may be slowed down by optimizing the ligands or functional groups^36^.

**Figure 4:**
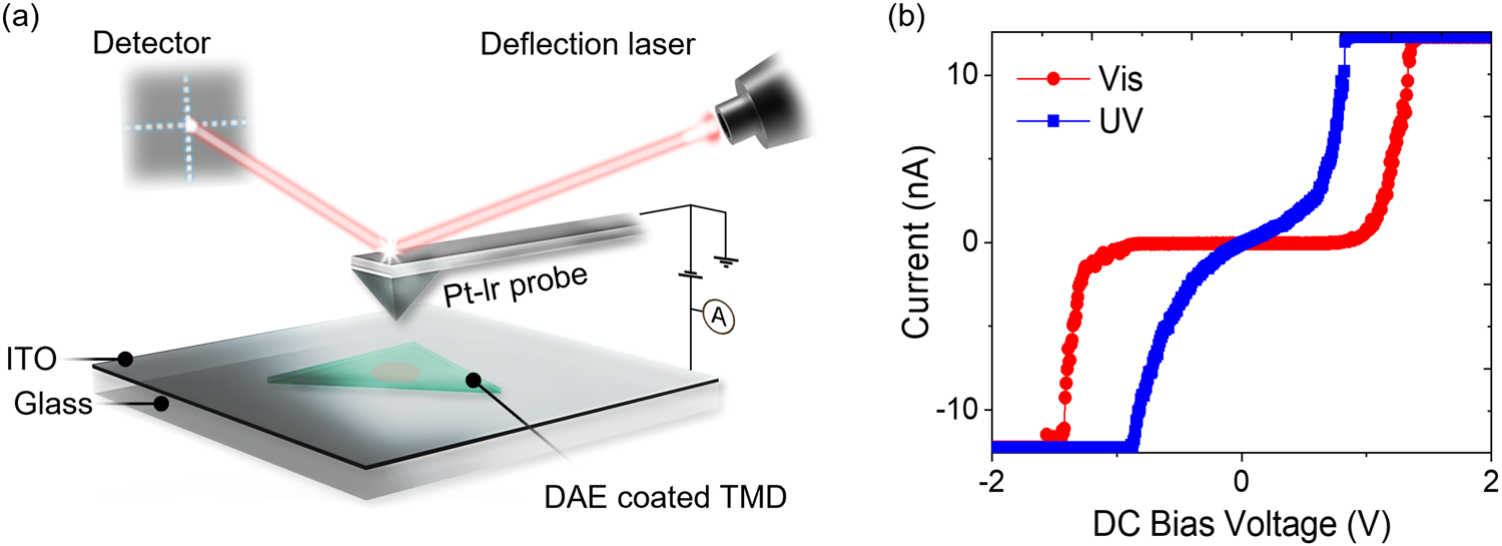
Conductive AFM of hybrid MoSe_2_/DAE after irradiation with visible and UV light. (**a**) Schematic of C-AFM where a TMD flake is exfoliated onto an ITO substrate and then coated with the DAE layer. (**b**) Current vs bias voltage plots measured by sweeping DC voltage from − 2 to 2 V. Note 12 nA are the maximum in the measured current window. After UV irradiation, the electron transfer between MoSe_2_ and DAE enhances current considerably.

UV irradiation to yield the ring-closed isomers will not only facilitate photoinduced electron transfer, suppressing excitonic recombination and leading to PL quenching, but also increase the conductivity in MoSe_2_. To probe this effect, we prepared DAE-coated MoSe_2_ flakes onto ITO glasses and examined them under C-AFM for current–voltage measurements. The samples were placed between the ITO and Pt-Ir probe as depicted in Fig. 4a, while applying a DC bias voltage of ± 2 V and limiting the current to ± 12 nA to protect the samples and the tip from localized charges. The results for open- and closed-ring isomers are shown in Fig. 4b. The UV-irradiated closed DAE sample exhibited a significantly higher current than the MoSe_2_ with open-ring DAE (under visible light) at the same applied potential. For example, the current after UV irradiation was greater than 12 nA in magnitude at ± 1 V, while it is nearly 0 nA under visible light. This increase in current is attributed to the UV-induced promotion of charge separation and transport due to the LUMO energy lowered by ~ 0.5 eV and below the CBM of MoSe_2_. In contrast, the LUMO energy of the open DAE is higher than the CBM and does not facilitate current generation, unlike the closed DAE. Since the VBM of MoSe_2_ is at approximately − 5.35 eV, it is lower than the work function of ITO (about − 4.75 eV)^51^. Therefore, a Schottky contact is established between MoSe_2_ and ITO, developing the Schottky barrier and yielding nonlinear current behaviors.

**Figure 5:**
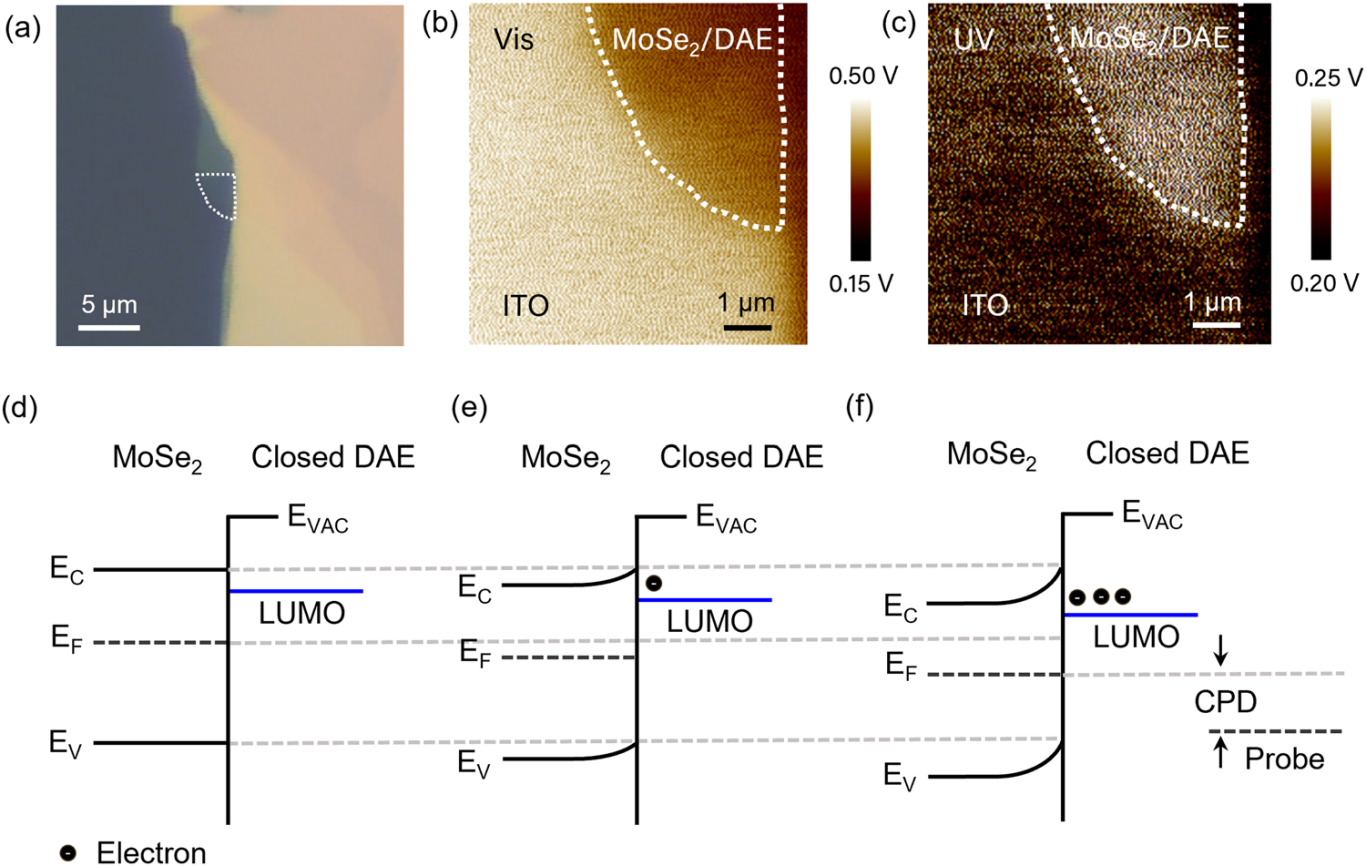
KPFM measurement of a MoSe_2_-DAE hybrid and a schematic of related band energy modulation. (**a**) Optical microscope image of MoSe_2_ exfoliated on an ITO glass substrate and measured surface potential of MoSe_2_/DAE following an exposure to (**b**) visible and (**c**) UV light. The average surface potentials are approximately 0.47 eV (Vis) and 0.22 eV (UV), from which we estimate work functions for MoSe_2_/DAE of about − 4.58 eV and − 4.83 eV, respectively. Band energy diagram (**d**) before contact between MoSe_2_ and closed-ring DAE, (**e**) shortly after establishing a hybrid interface, and (**f**) after charge transfer from MoSe_2_ to the DAE LUMO. Charge transfer at the hybrid interface leads to a band bending and downward shifts in the Fermi level of MoSe_2_.

When photoisomerization of DAE occurs, it alters the work function of MoSe_2_/DAE due to interlayer charge transfer between MoSe_2_ and DAE. We investigated the surface potential differences using KPFM to measure the change in work function. In the MoSe_2_/DAE structure, the energy level shift caused by UV exposure results in electron transfer to DAE, balancing the energy gap. The work function was calculated as follows^52,53^:

$$ \Phi_{{\text{S}}} = \, \Phi_{{{\text{tip}}}} + {\text{ CPD}} $$

where CPD stands for contact potential difference, and Φ_S_ and Φ_tip_ are the work functions of the sample and Pt-Ir probe (~ 5.05 eV)^19,54^, respectively. Figure 5 presents the energy potential maps of MoSe_2_/DAE. After exposure to visible light, an average surface potential of approximately 0.47 eV is observed, while UV-irradiated MoSe_2_/DAE displays a potential of ~ 0.22 eV. From this measurement, we have determined work functions under two distinct light conditions by subtracting surface potential values from the Pt-Ir probe potential. An approximate value of − 4.58 eV is obtained after visible light, while a work function under UV irradiation is about − 4.83 eV. These results confirm the interlayer charge transfer occurring in the hybrid structure. Figure 5d–f depict the energy diagram of MoSe_2_ in three different states: before, partially, and after reaching equilibrium at the hybrid interface. Electrons exiting the semiconductor layer create the Helmholtz layer, resulting in the upward bending of the band structure. The depletion layer is formed at the contact of the MoSe_2_ and DAE. As the transfer process progresses, the depletion layer thickens, leading to a decrease in the CBM and a lowering of the Fermi level of MoSe_2_ and the LUMO of closed-ring DAE^55^. This band modulation causes a reduction in CPD and increases the work function of the MoSe_2_/DAE structure. If the sample is exposed to visible light, the DAE will shift from a closed to an open isomer, causing the LUMO to change from − 4.31 eV to − 3.80 eV. Charge transfer behavior and work function will change accordingly. The interplay between photoisomerization, charge transfer, and surface potential alterations in MoSe_2_/DAE, as elucidated in this study, not only advances our understanding of hybrid materials but also opens new avenues for tailoring electronic properties in novel optoelectronic applications.

## Conclusions

In summary, we have demonstrated a drastic photo-modulation of the optoelectronic properties of TMD monolayers using photochromic DAE molecules. Photoisomerization of DAE within an ultrathin layer on MoSe_2_ flakes regulates photoinduced charge transfer at the hybrid interface. PL studies, conductive AFM, and KPFM measurements all confirm the proposed photoswitch scheme. Our findings prove the viability of charge transport modulation on 2D semiconductors by simple external light illumination. Given the extensive library of photochromic molecules, this approach may be applied to other 2D materials matching their electronic band structures. This will allow for novel designs of optoelectronic devices, offering simplicity in both preparation and device operation. We envision that this work will open new possibilities in 2D hybrid TMD systems.

### Supplementary Information

Supplementary Information.

## Data Availability

The datasets used and/or analyzed during this study will be available upon reasonable request.
