# Supplementary material for: Photoswitchable optoelectronic properties of 2D MoSe2/diarylethene hybrid structures
Source: Sci Rep. 2024 Mar 27;14:7325. doi: 10.1038/s41598-024-57479-z (PMC10973406; doi:10.1038/s41598-024-57479-z)
Supplement: Supplementary file 1 — Supplementary Information. [file 41598_2024_57479_MOESM1_ESM.pdf]

Supplementary Information

Photoswitchable Optoelectronic Properties of 2D $MoSe_2$/Diarylethene Hybrid Structures

Sewon Park[1†], Jaehoon Ji[1†], Connor Cunningham[2], Srajan Pillai[2], Jean Rouillon[3], Carlos Benitez-Martin[4], Mengqi Fang[5], Eui-Hyeok Yang[5], Joakim Andréasson[3], Jeong Ho You[2], and Jong Hyun Choi[1*]

[1] School of Mechanical Engineering, Purdue University, West Lafayette, Indiana 47907, United States
[2] Department of Mechanical Engineering, University of St. Thomas, St. Paul, Minnesota 55105, United States
[3] Department of Chemistry and Chemical Engineering, Chalmers University of Technology, SE-412 96 Gothenburg, Sweden
[4] Department of Chemistry and Molecular Biology, University of Gothenburg, SE-413 90 Gothenburg, Sweden
[5] Department of Mechanical Engineering, Stevens Institute of Technology, Hoboken, New Jersey 07030, United States

[†] Contributed equally to this work
[*] Corresponding author: jchoi@purdue.edu

Content

1. Synthesis and Characterization of DAE Molecules
2. Absorption Spectra of DAE
3. PL Reversibility Cycles of $MoSe_2$-DAE
4. DFT Calculation Results
5. References

1. Synthesis and Characterization of DAE Molecules

1.1 General methods and considerations for synthesis

Commercially available reagents and solvents were purchased and used as supplied. Reactions were monitored by analytical thin-layer chromatography (TLC) on a silica gel 60 F_{254}, Merck precoated silica gel plate, which were revealed by using a UV lamp. Flash-column chromatography was performed on Selekt flash chromatography systems (Biotage), using silica gel SNAP KP-Sil single-use column and solid deposition of the crude reaction.

NMR spectra were recorded at room temperature on a Bruker Avance NEO spectrometer, operating at 600 and 151 MHz for ^{1}H and ^{13}C, respectively. Chemical shifts were reported as values (ppm) with reference to the peak of DMSO-*d6*. Abbreviation for the ^{1}H NMR data were as follows: chemical shift δ, multiplicity (s = singlet, d = doublet, t = triplet, q = quartet, m = multiplet, br = broad), coupling constants J.

1.2 Synthesis and characterization

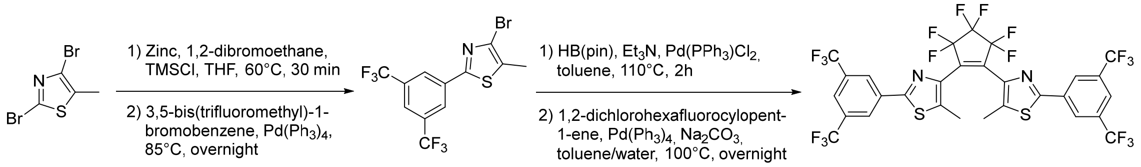

Scheme S1. Synthesis of DAE.

2-(3,5-bis(trifluoromethyl)phenyl)-4-bromo-5-methylthiophene

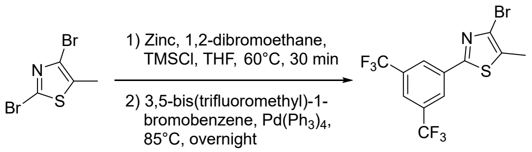

Knochel *et al.* modification[1] was adapted to a literature procedure[2] for the synthesis of 2-(3,5-bis(trifluoromethyl)phenyl)-4-bromo-5-methylthiophene. LiCl (99 mg, 2.34 mmol, 2.96 equiv.) was placed in an nitrogen-flushed flask and dried. Zinc (157 mg, 2.40 mmol, 3.04 equiv.) was added under nitrogen, and the resulting mixture was dried again. Then THF (2 mL) was added, and Zn was activated by 1,2-dibromoethane (20 µL, 0.23 mmol, 0.23 equiv.; heating to ebullition for 15 seconds) and TMSCl (10 µL, 0.08 mmol, 0.1 equiv.; heating to ebullition for 15 seconds). 2,4-dibromo-5-methylthiazole (203 mg, 0.79 mmol, 1.0 equiv.) in 1 mL of THF was added to the reaction mixture via a syringe, and it was stirred for 30 min at 60 °C. Then, 3,5-bis(trifluoromethyl)-1-bromobenzene (161 µL, 0.93 mmol, 1.18 equiv.) and $Pd(PPh_3)_4$ (95 mg, 0.08 mmol, 0.10 eq.) were added, and the mixture was refluxed at 85 °C overnight. After cooling to room temperature, it was diluted with Et_2O and filtered through Celite. Then, the organic phase was washed twice with aqueous HCl (1 M), aqueous saturated $NaHCO_3$ solution, and brine, and was dried over Na_2SO_4. After evaporation of the solvent the crude product was purified by flash chromatography (pentane/ DCM, 9:1) yielding 2-(3,5-bis(trifluoromethyl)phenyl)-4-bromo-5-methylthiophene. (229 mg, 0.59 mmol, 74 %) as a white solid. Proton NMR shown below agrees with previous reports.[2]

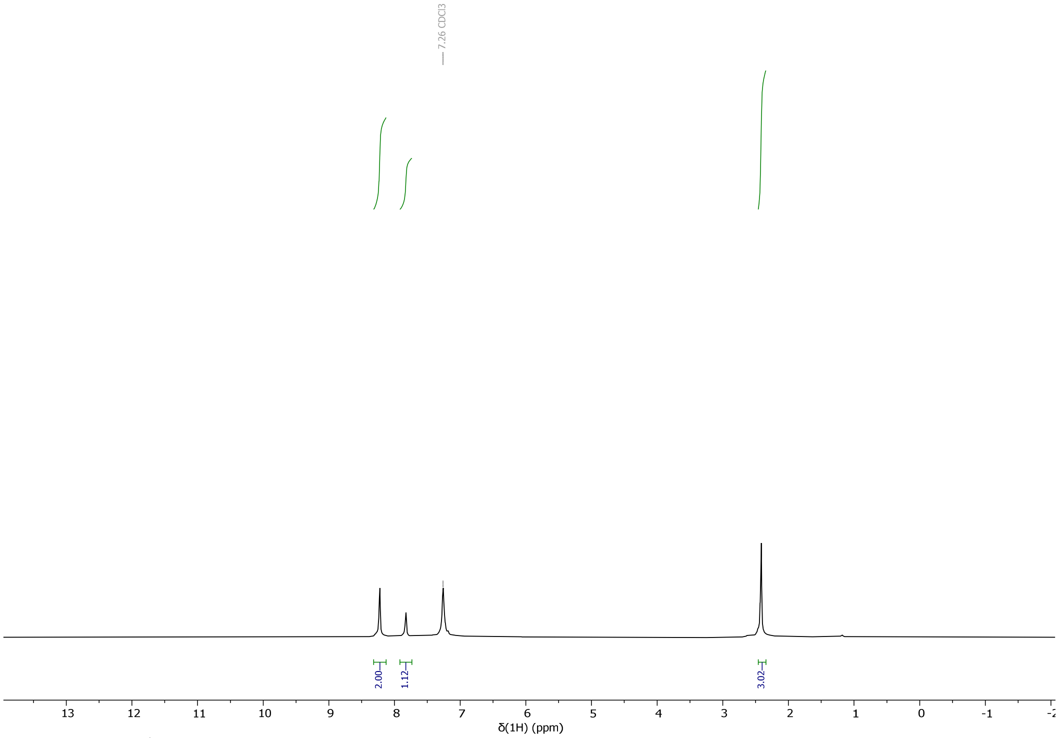

Figure S1. ^{1}H-NMR (600 MHz, $CDCl_3$): δ (ppm) = 8.22 (s, 2 H, CHar), 7.82 (s, 1 H, CHar), 2.41 (s, 3 H, CH3).

4,4'-(perfluorocyclopent-1-ene-1,2-diyl)bis(2-(3,5-bis(trifluoromethyl)phenyl)-5-methylthiazole) (**DAE**)

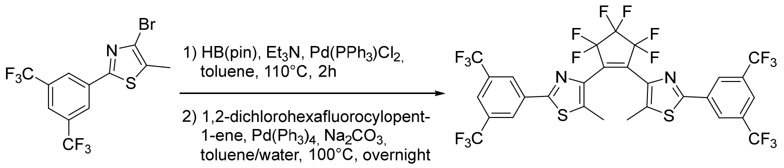

Desired DAE was prepared according to a known procedure.[2] Previously prepared bromothiazole derivative (230 mg, 0.59 mmol, 1.0 equiv.), triethylamine (493 µL, 3.54 mmol, 6.0 equiv.), and $Pd(PPh_3)_2Cl_2$ (23 mg, 0.03 mmol, 0.05 equiv.) were dissolved in 8 mL of dry toluene and the mixture was degassed by nitrogen bubbling. After the addition of pinacolborane (257 µL, 1.77 mmol, 3.0 eq.) the mixture was refluxed for 4 h at 120 °C. After cooling to 80 °C, an aqueous solution of Na_2CO_3 (2 M, 1 mL, 3.54 mmol) was added very slowly due to vigorous gas formation. Then, 1,2-dichlorohexafluorocylopent-1-ene (30 µL, 0.20 mmol, 0.33 eq.) and $Pd(PPh_3)_4$ (30 mg, 0.03 mmol, 0.03 eq.) were added and the mixture was stirred at 100 °C overnight. After cooling down to room temperature the mixture was extracted with ethyl acetate and the combined organic layers were washed with brine and dried over Na_2SO_4. Purification by flash chromatography (pentane/DCM 7:3) yielded the desired **DAE** (25 mg, 0.03 mmol, 16 %) as a white solid. Proton NMR in Figure S2 agrees with literature.[2]

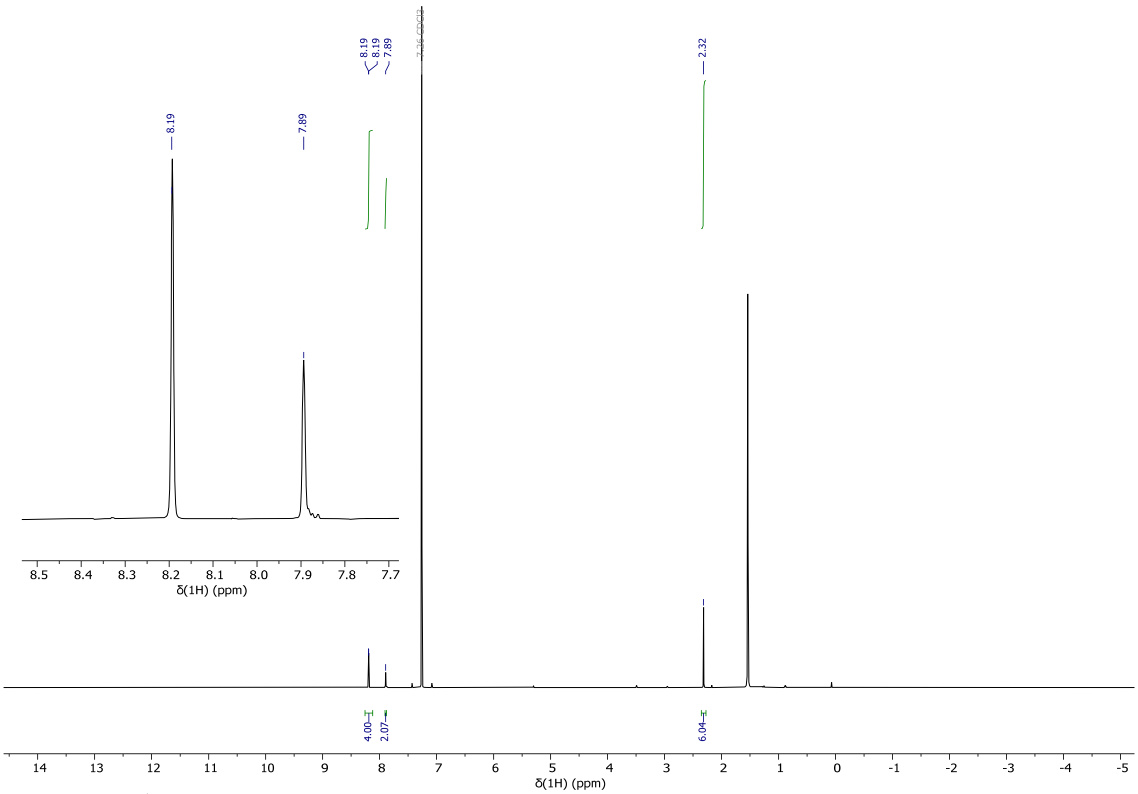

Figure S2. ^{1}H NMR (600 MHz, CDCl3): δ 8.19 (br s, 4 H, CHar), 7.89 (br s, 2 H, CHar), 2.32 (s, 6 H, CH3).

2. Absorption Spectra of DAE

The UV/Vis absorption spectra of DAE are measured using a Cary 5000 UV-Vis-nIR Spectrometer. Figure S3a shows the spectral evolution during UV irradiation at 312 nm. The rise of the absorption band in the visible region clearly shows that the UV light is triggering the isomerization of the open- to the closed isomeric form. UV exposure for 2 minutes was sufficient to reach near the photostationary distribution.

To demonstrate the reversibility between closed and open isomers, visible light was applied to DAE samples using a Newport 67005 light source with a 530 nm long-pass filter in a strictly controlled environment to avoid exposure to external light. The solution previously exposed to 2 minutes of UV light was irradiated by the visible light and the absorption spectra was monitored with time. The absorbance data presented in Figure S3(b) clearly shows a reversal of the isomerization process to re-form the main part of the sample to the initial open isomer within 10 minutes.

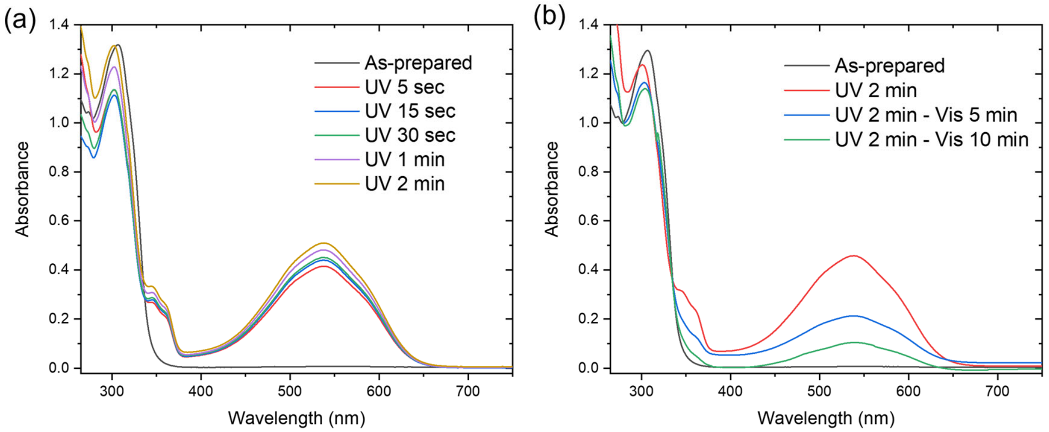

Figure S3. UV/Vis absorption spectra of (a) DAE molecules in chroloform solution (~0.02 mg/mL) exposed to varying doses of UV irradiation and (b) samples subjected to 2 minutes of UV irradiation followed by visible (Vis) irradiation.

3. PL Reversibility Cycles of $MoSe_2$-DAE

Figure 3(e) shows a drastic PL quenching (approximately by ~80%) due to photoswitching from open to closed DAE. We find that several other $MoSe_2$-DAE samples also demonstrate about 80% on average. Next, we investigated the reversibility of $MoSe_2$-DAE interactions with confocal Raman spectroscopy (Renishaw inVia). Ten complete cycles were executed with alternating UV/Vis irradiation. The results are presented in Figure S4 which is also summarized in Figure 3(f). There are moderate changes in PL intensities after multiple irradiation cycles due to side reactions.[2, 3] Nonetheless, it is evident that the photoswitching between open and closed DAE by UV/Vis irradiation can drastically modulate the optoelectronic properties of $MoSe_2$.

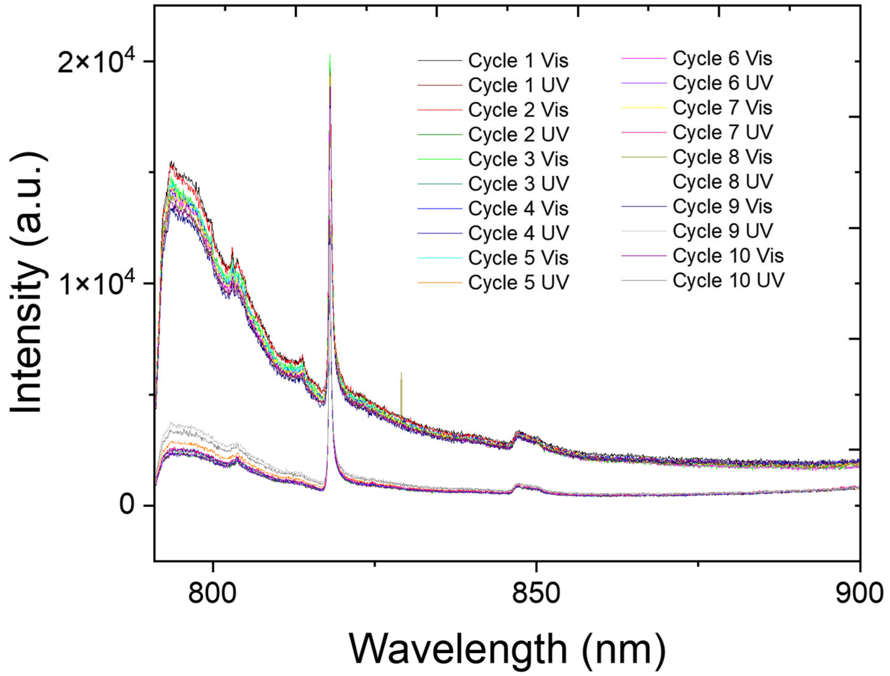

Figure S4. Reversibility of the PL emission signatures under alternating UV/Vis irradiation. The results from ten successive cycles confirm the reversible behavior of the hybrid structure.

4. DFT Calculation Results

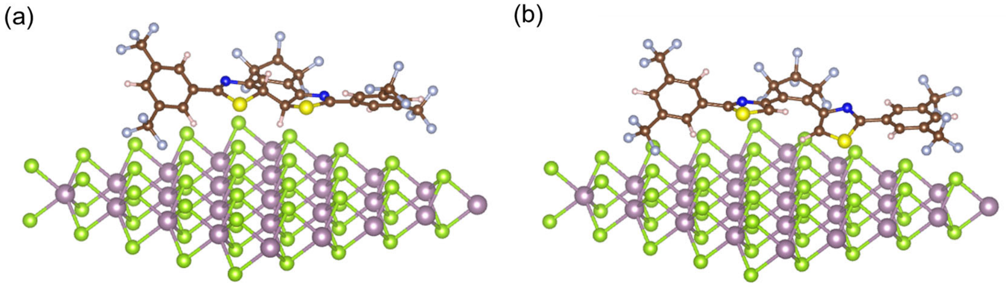

Figure S5. Relaxed structures of hybrid DAE-$MoSe_2$. (a) Closed and (b) open DAE isomers.

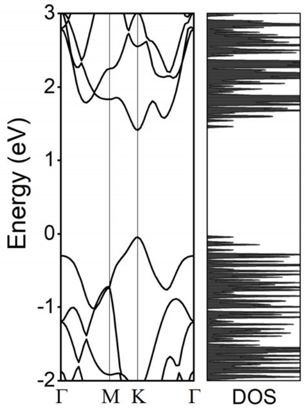

Figure S6. DFT-calculated band structure and density of electronic states of a (1×1) $MoSe_2$ monolayer. A vacuum of ~15 Å was added. A 10×10×1 Γ-centered **k**-point mesh was used for ionic relaxation and a 20×20×1 mesh for electronic calculations. A direct bandgap is observed at **K**. The VBM energy level is set to be zero.

6. References

1. Sase, S.; Jaric, M.; Metzger, A.; Malakhov, V.; Knochel, P., One-Pot Negishi Cross-Coupling Reactions of In Situ Generated Zinc Reagents with Aryl Chlorides, Bromides, and Triflates. *Journal of Organic Chemistry* **2008,** *73*, 7380-7382.
2. Herder, M.; Schmidt, B. M.; Grubert, L.; Pätzel, M.; Schwarz, J.; Hecht, S., Improving the fatigue resistance of diarylethene switches. *Journal of the American Chemical Society* **2015,** *137*, 2738-2747.
3. Jeong, Y.-C.; Park, D. G.; Lee, I. S.; Yang, S. I.; Ahn, K.-H., Highly fluorescent photochromic diarylethene with an excellent fatigue property. *Journal of Materials Chemistry* **2009,** *19*, 97-103.
